# Feasibility and Outcomes of an Internet-Based Mindfulness Training Program: A Pilot Randomized Controlled Trial

**DOI:** 10.2196/mental.5457

**Published:** 2016-07-22

**Authors:** Pia Kvillemo, Yvonne Brandberg, Richard Bränström

**Affiliations:** ^1^ Department of Clinical Neuroscience Karolinska Institutet Stockholm Sweden; ^2^ Department of Oncology-Pathology Karolinska Institutet Stockholm Sweden

**Keywords:** mindfulness, Internet-based intervention, Internet, usability, acceptability, feasibility, randomized controlled trial

## Abstract

**Background:**

Interventions based on meditation and mindfulness techniques have been shown to reduce stress and increase psychological well-being in a wide variety of populations. Self-administrated Internet-based mindfulness training programs have the potential to be a convenient, cost-effective, easily disseminated, and accessible alternative to group-based programs.

**Objective:**

This randomized controlled pilot trial with 90 university students in Stockholm, Sweden, explored the feasibility, usability, acceptability, and outcomes of an 8-week Internet-based mindfulness training program.

**Methods:**

Participants were randomly assigned to either an intervention (n=46) or an active control condition (n=44). Intervention participants were invited to an Internet-based 8-week mindfulness program, and control participants were invited to an Internet-based 4-week expressive writing program. The programs were automated apart from weekly reminders via email. Main outcomes in pre- and postassessments were psychological well-being and depression symptoms. To assess the participant’s experiences, those completing the full programs were asked to fill out an assessment questionnaire and 8 of the participants were interviewed using a semistructured interview guide. Descriptive and inferential statistics, as well as content analysis, were performed.

**Results:**

In the mindfulness program, 28 out of 46 students (60%) completed the first week and 18 out of 46 (39%) completed the full program. In the expressive writing program, 35 out of 44 students (80%) completed the first week and 31 out of 44 (70%) completed the full program. There was no statistically significantly stronger intervention effect for the mindfulness intervention compared to the active control intervention. Those completing the mindfulness group reported high satisfaction with the program. Most of those interviewed were satisfied with the layout and technique and with the support provided by the study coordinators. More frequent contact with study coordinators was suggested as a way to improve program adherence and completion. Most participants considered the program to be meaningful and helpful but also challenging. The flexibility in performing the exercises at a suitable time and place was appreciated. A major difficulty was, however, finding enough time to practice.

**Conclusions:**

The program was usable, acceptable, and showed potential for increasing psychological well-being for those completing it. However, additional modification of the program might be needed to increase retention and compliance.

**ClinicalTrial:**

ClinicalTrials.gov NCT02062762; https://clinicaltrials.gov/ct2/show/NCT0206276 (Archived by WebCite at http://www.webcitation.org/6j9I5SGJ4)

## Introduction

Structured group-based meditation interventions, such as the Mindfulness-Based Stress Reduction (MBSR) program [1], have increasingly been used to alleviate stress in individuals over the last several decades. The concept of mindfulness has been defined as “the awareness that emerges through paying attention on purpose, in the present moment, and nonjudgmentally to the unfolding of experience moment by moment” [2]. In mindfulness-based programs, a number of specific meditation and yoga exercises are used to develop and increase the ability to be mindful in the present moment. The MBSR program was originally developed for individuals with somatic disorders [3], but over the years the effects of mindfulness-based programs have been evaluated in a large number of studies for various population groups and for different symptoms and conditions. A meta-analysis examining the effects of mindfulness training in nonclinical settings on a wide variety of outcomes showed small to medium effect sizes for anxiety, stress reduction, and psychological well-being [4].

The Internet provides an opportunity to deliver evidence-based psychosocial programs to a broader range of individuals and a possibility to disseminate interventions targeting stress and stress-related problems to large groups [5-8]. An increasing proportion of the global population has access to the Internet—more than 70% of people in Europe, nearly 90% in North America, and 95% in Sweden are Internet users [9]. Internet-based programs can be cost efficient [10], and the stigma that might be associated with some face-to-face consultations regarding mental disorders can be avoided [6]. A growing number of treatment programs are Internet-based, targeting many stress-related and mental conditions and behaviors such as smoking, insomnia, and depression [11-13]. An increasing number of studies of patients with various mental disorders have shown the positive effects of Internet-based programs [14-18]. Several of these programs have been based on or included mindfulness techniques. The effects of specific mindfulness-based programs delivered through the Internet have been promising regarding the improvement of mental states such as stress, anxiety, and depression [19-23], and results apply to persons with psychological symptoms of stress or distress [19], as well as to persons without previously known mental health problems [21,24], including students [23].

Several studies have explored the experiences of Internet-based interventions among users, although few of them have investigated participant perceptions of specific mindfulness-based programs. In one randomized controlled trial (RCT) assessing the feasibility of an Internet-based mindfulness program for stress management in the Unites States, participants were asked to complete a questionnaire about overall feedback on the intervention and reasons for early study or program termination [22]. About 25% of those who completed the baseline questionnaire responded to the feedback questionnaire. Of those who gave feedback, 45% found the overall program to be very or extremely helpful, 35% somewhat helpful, and 19% little or not at all helpful. The most common reason for leaving the program was that the participant was too busy. The second most common reason for termination was technical or access problems. In an American interview study assessing participant experiences of a mindfulness-based intervention delivered through the Internet for reducing residual depression symptoms and preventing relapse, participants reported on a perceived lack of support during the program but appreciated the flexibility of completing weekly sessions according to their own schedule [25].

This randomized controlled pilot trial examined the feasibility, usability, acceptability, and outcomes of a newly developed 8-week Internet-based mindfulness training program among Swedish students. Feasibility was examined by determining the degree to which participants were engaged in and completed the program. Usability and acceptability were assessed using postintervention questionnaires and semistructured interviews of experiences at completion of the program. Outcomes were examined by comparing pre- and postassessments of psychological well-being and depression symptoms both within the intervention group and between those participating in the mindfulness training program and those randomized to an active control condition. This broad approach of evaluation aims to expand the knowledge about the potential of Internet-based self-administrated mindfulness-based programs to improve mental health status.

## Methods

### Participants and Procedures

Study participants were recruited between December 2013 and March 2014 by advertising at different university campuses in Stockholm, Sweden. The study was open to students aged 18 years and older with access to a computer and an email address. Students interested in participation phoned or sent an email to the study coordinators to receive additional information about the study design. Participants were randomized to either the intervention group (Internet-based mindfulness training program) or an active control group (Internet-based expressive writing program) on a rolling basis using a random sequence of numbers. One of the authors generated the sequence of numbers using SPSS statistical analysis software (IBM Corp) and another author enrolled and assigned participants in the order they were recruited. All participants in both groups were asked to fill out a questionnaire before and after the program and complete weekly assessments. Participants who did not respond to the weekly assessments were reminded by email. The completion of a week’s training was not time-limited, but the next set of program material was only made available after the participant completed the previous training and assessment. Those who did not respond to the reminder or did not fill out the weekly assessments marking continued participation were not asked to give a reason for discontinuation or noncompliance. All participants completing the Internet-based programs were compensated in an amount of SEK 500 (about $54).

### The Internet-Based Mindfulness-Based Intervention

The mindfulness training program developed for this study was a modified version of the group-based mindfulness program developed by Jon Kabat-Zinn [3,26]. The adaptation of the program to an Internet environment was based on experiences gained from other Internet-based programs [27-32]. Participants were given access to the course platform by logging in and using an individual password. The program consisted of 8 weekly modules including information about the theoretical foundations of mindfulness regarding relaxation, meditation, and the body-mind connection. Each weekly module consisted of a few pages of text (ie, the lecture) and a set of exercises. All formal exercises described in the standards of practice of MBSR from the University of Massachusetts Medical School Center for Mindfulness in Medicine [26] (body scan meditation, hatha yoga, sitting meditation, and walking meditation) as well as the informal exercises awareness of pleasant and unpleasant events, awareness of breathing, and deliberate awareness of routine activities and events were included in the program (see Table 1).

**Table 1:** Program content.

	Topic of lecture	Formal exercises	Informal exercises
Week 1	Mindfulness: benefits to quality of life and health	Introduction of: – Mindful breathing – Body scan meditation	Introduction of: – Deliberate awareness of routine activities and events such as waking up, eating, taking a shower, driving, awareness of interpersonal communications
Week 2	Cultivation of mindful attitudes	Continued practice of introduced exercises	Continued practice of introduced exercises
Week 3	The desire to keep or avoid	Introduction of: –Lying yoga Continued practice of introduced exercises	Introduction of: – 3-minutes meditation: ○Step 1: Becoming aware ○Step 2: Gathering and focusing attention ○Step 3: Expanding attention Continued practice of introduced exercises
Week 4	Mindfulness and stress	Continued practice of introduced exercises	Continued practice of introduced exercises
Week 5	Relations and social context	Introduction of: – Standing yoga – Sitting meditation – Walking meditation Continued practice of introduced exercises	Continued practice of introduced exercises
Week 6	Automatic thoughts	Continued practice of introduced exercises	Introduction of: – STOP: A Short Mindfulness Practice enabling distancing from instant feelings Continued practice of introduced exercises
Week 7	Sleep and Mindfulness	Encouragement to train without audio files and experiment with different combinations of exercises	Introduction of: – Short exercise to facilitate falling asleep Continued practice of introduced exercises
Week 8	No lecture	Encouragement to again use audio files and choose preferred exercises	Continued practice of introduced exercises

The main departures in the content of our self-administrated program from MBSR as described in the standards of practice from the University of Massachusetts Medical School [26] were (1) no face-to-face sessions, (2) no group dialogue or contact between participants, (3) no all-day silent retreat during the sixth week, and (4) no introduction of self-evaluation instruments at the end of the course. Participants could navigate within each module in the texts by clicking on navigational icons. Audio files, 15 or 30 minutes in length, were provided to facilitate daily practice of formal exercises. The participants were encouraged to practice 30 to 45 minutes a day, continuously or throughout the day, 6 to 7 days per week. The program also included short-duration meditation and exercises aimed to promote the integration of mindful awareness into everyday activities. New exercises were introduced gradually during the first 5 weeks, including mindful breathing and body-scan meditation in the first and second weeks, lying yoga in the third and fourth weeks, and standing yoga, sitting meditation, and walking meditation in the fifth week. In weeks 6 to 8, participants could choose which exercises to perform. None of the exercises required a high degree of physical strength or agility. Participants were presented with alternative ways of doing the exercises if needed, like choosing other positions, to ensure their safety. The study coordinator supported participants by sending weekly messages informing them that a new week’s text and exercises were available. The study coordinator could monitor each participant’s log-in history and send extra reminders to participants who did not visit the platform for 14 days or longer. The participants could contact the study coordinators through the program platform or by sending an email or making a phone call.

### The Internet-Based Expressive Writing Intervention

Participants in the Internet-based expressive writing program were asked to write about stressor-related emotions and thoughts for 20 minutes on 4 occasions spread out over approximately 4 weeks. This procedure is drawn from work by Pennebaker and colleagues and has been tested across dozens of RCTs [33]. In this study, the standardized procedure by Pennebaker was complemented with an additional writing instruction. In addition to writing about stressor-related emotions, participants were asked to write for 10 minutes using a positive prompt following the first writing assignment. Examples of positive prompts are: “What has become better since . . .,” “What personal strengths helped you deal with . . .,” and “What makes you feel hopeful about the future?” The exercises were made available on an Internet-based platform participants could access by logging in using an individual password. Participants could make contact with the study coordinators through the program platform or by sending an email or making a phone call.

### Internet-Based Measurements at Baseline and Postintervention

#### Participant Characteristics

Participants were asked to complete a baseline questionnaire which included questions on age, gender, level of education, and living situation. This was done in connection to the obtaining of baseline measures of the outcome variables.

#### Outcome Variables

Psychological Well-Being is a questionnaire measuring 6 dimensions: environmental mastery, self-acceptance, positive relations with others, purpose in life, personal growth, and autonomy. It has been extensively used and has shown acceptable factor structure and validity [34]. In this study, an overall total measure of psychological well-being was used. The alpha coefficient of the scale was .82 in our sample.

The Center for Epidemiologic Studies Depression Scale is a 20-item scale measuring depression symptoms in nonpsychiatric populations [35]. The alpha coefficient of the scale was .90 in our sample.

#### Weekly Follow-Up Assessments

After each week, the participants in the mindfulness intervention group were asked to answer questions about how much time they had spent on exercises during the previous week. Participants in both groups were also asked to respond to a number of questions assessing potential mediators of intervention effect: mindfulness skills after weeks 2, 4, 6, and 8 and positive/negative affect after weeks 1, 3, 5, and 7. For the analyses in this paper, the time and frequency of practice is reported, but no analyses of the mediation was performed.

#### Evaluations of the Program

At the postassessment follow-up, respondents were asked to answer 7 questions on a scale of 1 to 9, where 1 indicates “not at all” and 9 indicates “very,” to evaluate their overall experiences of the programs. The questions were based on research into factors considered to be important for participation in Internet-based programs (ie, how well the intervention is received and perceived by users) [22,29,31] and included: How meaningful do you think the program was? How successful do you think the program was in improving your way of being? How likely is it that you would recommend the program to someone in the same situation as you? How emotionally demanding do you think the program was? On the whole, how challenging do you think the program was? If you had known as much about the program before starting as you know today, how likely would it have been that you would still have participated? Do you feel that your ability to reach mindful awareness has changed during the program? Items rated 1 to 3 points were considered to have a low value to participants; items rated 4 to 5, middle value; and items rated 7 to 9, high value.

### Statistical Analysis

Baseline characteristics of the sample, stratified by experimental group, were examined to ensure that key variables were evenly distributed by randomization using Student *t* tests. The most specific test of the hypothesis as a whole was the difference between groups on the contrast between baseline and follow-up. This was tested using multivariate repeated-measures analyses of variance (MANOVA) with baseline and follow-up scores as dependent variables. Time and group (mindfulness vs expressive writing) were entered as factors in the analyses. In addition to the MANOVA, inferential analyses were conducted to examine pre- and postintervention change for each dependent variable, stratified by randomization condition, using independent sample *t* tests. Cohen's *d* effect size for within and between group differences was calculated based on the difference between group means on baseline and follow-up change scores. The denominator was based on the pooled standard deviation (SD) at baseline and follow-up adjusted for different sample sizes [36]. All tests of intervention effects were done with intention-to-treat analyses with missing data at follow-up imputed according to last-observation-carried-forward strategy, meaning that some baseline values were used as postintervention values. All tests of significance were two-tailed. Analyses were performed in SPSS.

### Semistructured Telephone Interviews Postintervention

To get information about participant perceptions of the mindfulness training program, telephone interviews were conducted with a small number of participants shortly after program completion. The focus of the interviews was the participant experience of the techniques, information, instructions, and exercises, as well as the perceived support received from the study coordinators. The first students who completed the mindfulness program were asked to participate in an interview. One of the study coordinators (PK) conducted all interviews using a semistructured guide with open-ended questions along with probing statements. The interview guide reflected issues that have previously been shown to influence participant perceptions of Internet-based programs (eg, if the program was easy to use [32], how it was perceived in terms of reliable information and professional appearance, and challenges in carrying out the exercises [22,27-30]). The interviews were 15 minutes long on average and recorded with the permission of the interviewees. After 6 to 7 interviews, no new information seemed to appear, and the interview process was discontinued when 8 participants had been interviewed. The interview questions were as follows: Do you want to say something in general about the program and your experiences of it? How did you experience the Internet technique by which the program was delivered? How did you experience the information in the program? How did you experience the interaction with the study coordinators? How did you experience the training? What is your view on the items in the questionnaires? Do you have any suggestions on how the program could be improved? The audio records were transcribed verbatim after the interview process was completed.

### Content Analysis of Semistructured Interviews

Qualitative content analysis was used to analyze the interview data [37]. The analysis was to a large extent deductive, since the interview questions initially directed the analysis. Unexpected content was also taken into account in order to refine and extend the understanding of the material. The approach most closely resembling ours has been described as directed content analysis [38]. A team-based approach was used for developing codes and coding the narratives [39]. All interviews were initially coded by researcher PK, who developed a coding scheme by identifying meaningful units that could be grouped into categories, as exemplified in Table 2. To assess the reliability of the coding [40], an independent recoding was undertaken by researcher YB using the original coding scheme. A high degree of agreement between coders was obtained with disagreements resolved through discussion.

**Table 2:** Example of analysis.

Meaning unit	Condensed meaning unit	Code	Subcategory	Category
I think that the layout was very good. The way you clicked and came forward. You click on one side and then you move on in a logical sequence. And then all the exercises, and after that the questions before starting the next week.	The user perceived the platform as easy to navigate.	The platform was perceived as easy to navigate.	Program-related facilitators	Usability

### Ethical Considerations

All procedures were performed in accordance with the ethical standards of the institutional and/or national research committees, the 1964 Helsinki Declaration and its later amendments, or comparable ethical standards. Informed consent was obtained from all the individual participants included in the study. The study was approved by the Ethics Committee of the Karolinska Institutet (No. 2010/1407-31).

## Results

### Enrollment, Follow-Up, and Demographics

**Figure 1:**
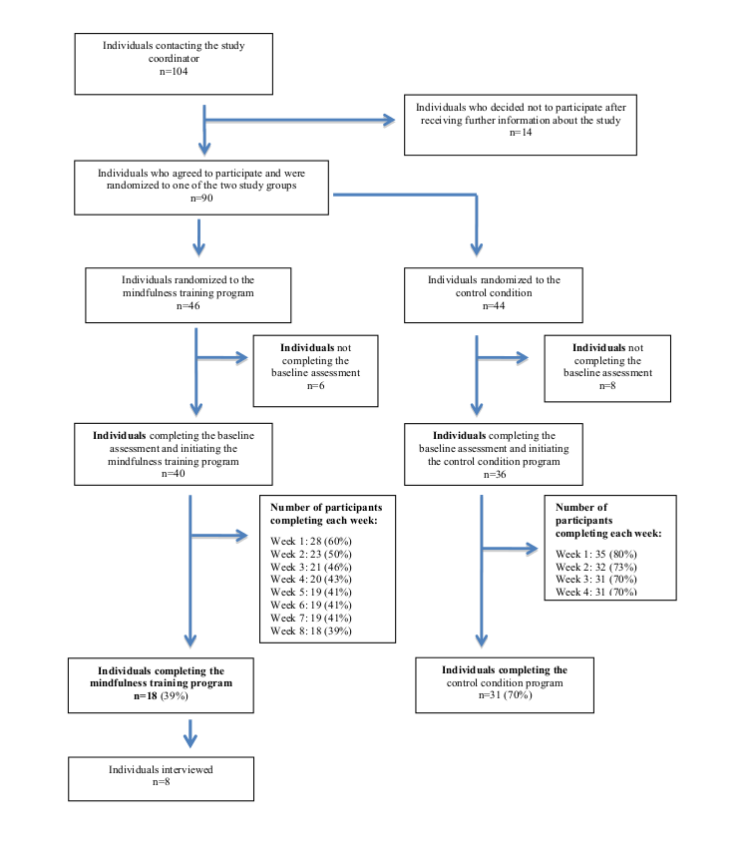
Flowchart showing enrollment and number of participants at each phase of the study.

A total of 104 students contacted the study coordinators requesting more information about the study or expressing an interest in participating with 90 students deciding to participate. Some students left the trial before completing the baseline questionnaire and initiating the program (6 were randomized to the mindfulness training program and 8 to the control condition). A flowchart showing enrollment and number of participants completing each week of the programs is presented in Figure 1.

Of those randomized to the mindfulness training program (n=46), 40 individuals (30 women and 10 men) began the program and 39% (18/40, 16 women and 2 men) completed it. A total of 22 participants (14 women and 8 men) did not complete the postintervention questionnaire. The median age of the completers was 25 years (range 18-45), and the mean age was 29 years. Corresponding figures for the 22 participants who did not complete all 8 weeks of the mindfulness program was 22 years (range 19-37) and 24 years, respectively. On average, participants practiced 3.6 days per week. Even though the reason for participants to leave the program was not systematically assessed, half of those who terminated the program before completion stated an explanation for leaving by email to the study coordinator. Of these, 9 mentioned lack of time, one had technical problems with the computer at home, and one participant referred to changed circumstances. Of the 18 who completed the mindfulness program, 8 (7 women and 1 man) were interviewed.

Of the 44 students randomized to the expressive writing intervention, 36 (26 women and 10 men) began the program and 31 (70%, 23 women and 8 men) completed it. A total of 5 who started the program (3 women and 2 men,) did not complete the postintervention questionnaire.

Those who completed the programs were significantly older than those who did not (*t*=2.22, *P*=.03). There was, however, no significant difference in gender, level of education, or income.

### Effects of the Mindfulness Training Program

There were no statistically significant differences between the intervention and control groups concerning baseline scores on psychological well-being (*t*=−1.07, *P*=.29) and depression symptoms (*t*=0.69, *P*=.49).

The MANOVA analyses with baseline and follow-up on psychological well-being and depression symptoms showed no significant time × group (intervention vs active comparison) interaction (*F*_2,73_=0.18, *P*=.83, partial *η*^2^=0.005), indicating that there was no significantly stronger intervention effect for the mindfulness intervention compared to the expressive writing intervention. No significant intervention effect was found in the overall test, but separate within-group comparisons are presented in Table 3. In these within-group comparisons, participants in the Internet-based mindfulness training program had a statistically significant increase in psychological well-being over time with a small effect size (*d*=0.2). No statistically significant change over time appeared for depression symptoms. Participants in the control condition did not report any statistically significant pre- and postassessment changes on the two outcomes.

**Table 3:** Results of within-group *t* test of change on outcome variables.

		Intervention condition (n=40) Internet-based mindfulness program mean (SD)	*P* value	Active control condition (n=36) Internet-based expressive writing program mean (SD)	*P* value
Psychological well-being					
	Baseline	60.0 (8.7)		62.7 (13.0)	
	Postintervention follow-up	62.0 (10.1)	.04	64.1 (11.8)	.11
Depression symptoms					
	Baseline	20.3 (10.4)		18.7 (9.9)	
	Postintervention follow-up	18.2 (9.6)	.08	17.5 (9.8)	.30

### Participant View of the Program in the Postintervention Evaluation

The results from the postintervention questionnaire are presented in Table 4. Overall, the participants in the intervention group were satisfied with the program, and all of them thought it was meaningful to some degree. All but one participant found the program to some extent successful in improving their way of being but most of them also found it challenging. Participants in the control group seemed to find their program less challenging as compared to the participants in the intervention group.

**Table 4:** Participant responses to the postintervention questionnaire.

		Not at all n (%)	Some n (%)	A lot n (%)	*P* value
**How meaningful do you think the program was?**					.11
	Mindful	0 (0)	7 (39)	11 (61)	
	Control	4 (13)	16 (52)	11 (36)	
**How successful do you think the program was in improving your way of being?**					.21
	Mindful	1 (6)	13 (72)	4 (22)	
	Control	8 (26)	18 (58)	5 (16)	
**How likely is it that you would recommend the program for someone in the same situation as you?**					.35
	Mindful	1 (6)	8 (44)	9 (50)	
	Control	6 (19)	14 (45)	11 (36)	
**How emotionally demanding do you think the program was?**					.93
	Mindful	8 (44)	7 (39)	3 (17)	
	Control	14 (45)	13 (42)	4 (13)	
**On the whole, how challenging do you think the program was?**					<.001
	Mindful	1 (6)	5 (28)	12 (67)	
	Control	16 (52)	15 (48)	0 (0)	
**If you had known as much about the program before starting as you know today, how likely would it have been that you would have still participated?**					.28
	Mindful	0 (0)	9 (50)	9 (50)	
	Control	4 (13)	13 (42)	14 (45)	
**Do you feel that your ability to reach mindful awareness has changed during the program?**					
	Mindful	0 (0)	6 (33)	12 (67)	
	Control	—	—	—	

### Results From Content Analyses of the Telephone Interviews

Two main categories and seven subcategories were identified in the interview material. In the category *usability* —defined in terms of the extent to which applications were easy to learn, error-tolerant, efficient, effective, and engaging [32]—the subcategories were program-related facilitators, program-related deficiencies, and proposals for improvement. We define *acceptability* in terms of how well the intervention was received by users, met their needs, and how challenging it was perceived [31]; its subcategories were personal facilitators, personal barriers, positive experiences, and negative experiences. The reason for including proposals for improvement in the first category was that the proposals concerned factors closely related to the way the program was designed and organized and thus relating to how easy it was to use. In what follows, the results of the content analyses are presented under headings that accord to the names of the subcategories.

### Program-Related Facilitators

The salient topics in this subcategory are positive or satisfactory experiences of technique, web layout, information, questionnaires, and support from study coordinators. Most of the participants were satisfied with the technique and web layout and reported no problems navigating and downloading files.

I think that the layout was very good, the way you clicked and came forward. You click on one side and then you move on in a logical sequence. And then all the exercises. And after that, the questions before starting the next week.

Woman, 26 years

I had no problems with the technology and stuff, with audio files and so on.

Woman, 32 years

Most of the participants perceived the content of the information texts and audio files as informative and enjoyable. The amount of information was also regarded as suitable. The differing lengths of the guiding audio files were appreciated.

I thought it was a very good way to get some background information every week. I thought the texts were just the right length. The material was interesting to read.

Woman, 23 years

Opinions regarding the weekly questionnaires varied among those interviewed. Some participants made both positive and negative statements about them. Positive statements concerned, for example, clarity and suitable scales. The communication with study coordinators and the weekly reminders were perceived as positive and supportive by several of the participants.

Yes, it has been great. The weeks go so damn fast, so without the emails, I probably would have forgotten about it altogether.

Woman, 25 years

Participants felt that they got answers quickly when they emailed questions to the study coordinators. The weekly reminders made it easier for them to remember to practice.

### Program-Related Deficiencies

The salient topics in this subcategory are negative or unsatisfactory experiences of web layout, information, and questionnaires. Although most of the participants expressed satisfaction with the information content of the program, some highlighted deficiencies in the texts, focusing on spelling, grammar, and lengthy sentences.

Yes, there was a typo here and there and, yes, maybe there was a sentence structure or two, which I couldn’t understand. But there were some typos, I reacted to them [. . .] there were unfinished sentences that were difficult to understand.

Woman, 26 years

The organization of audio files in the weekly modules was perceived as confusing by one of the respondents. In addition, some participants had complaints about the questionnaires. Most of these were about repetitive questions and difficulties in finding a suitable answer.

### Proposals for Improvement

This category comprises explicit proposals for improvements of the program mentioned by the interviewees. Some participants suggested improvements regarding the interaction with study coordinators and technical and layout issues. One of the respondents said that more emails reminding participants to exercise might facilitate completion of the program. Another suggestion was to arrange a meeting with the study coordinators and other participants at the beginning of the program in order to facilitate participation in and completion of the program. It was also suggested that a phone call from the study coordinators at the beginning of the program would increase the completion rate. Other suggestions were related to technical improvements, such as providing access to a special smartphone app where the participants could log their training. A reorganization of the audio files was proposed as was the provision of some kind of bank with questions from other participants, with related answers from the study coordinators.

### Personal Facilitators

The topics included in this subcategory are about circumstances connected to the participants’ personal situations that contributed to facilitating implementation of the program. Some of them were related to attitudes or motivation, like curiosity and personal interest, and others to more practical or technical conditions, like having the opportunity to download files onto a phone and listen to them on the way to work.

Well, I have a positive attitude towards it because I have practiced mindfulness before, privately on my own, in different ways [. . .] I am very curious and interested.

Woman, 32 years

### Personal Barriers

The topics in this subcategory are about circumstances connected to the participants’ personal situations that impeded implementation of the program. The absolutely dominant topic was that participants had difficulties in getting the training to fit into their daily lives and in finding the time to practice.

During that time, I was preparing for an exam and it was a bit hard and challenging to find the time to perform these exercises, and it was a little more stressful than I thought. I needed time, and more time, and everything.

Woman, 28 years

At the beginning I was more careful to ensure that it was 45 minutes every day, but then sometimes it became only 30 minutes. Then there were some days when my schedule didn’t work out, and I didn’t have time for anything at all.

Woman, 25 years

A couple of other issues were also mentioned as impeding the training. One of the participants had technical problems with the computer at home, and another lived together with a partner in a small flat and therefore had difficulties finding an undisturbed environment to practice.

### Positive Experiences

Statements reflecting satisfaction that are not closely related to the technical and pedagogic functioning of the program were categorized as positive experiences. A prominent topic in this subcategory is appreciation of the opportunity to choose between exercises and time to practice.

The advantage is that it has been great to be able to do it just when you feel like it, that you don’t need to fit in with a time.

Woman, 23 years

A number of participants also mentioned positive experiences from the training itself, like getting help to relax and calm down. The facts that participation was anonymous and that the exercises were performed at home were also mentioned as positive, as well as the experience of being in a permissive condition.

### Negative Experiences

Statements reflecting dissatisfaction not closely related to the technical and pedagogic functioning of the program were categorized as negative experiences. The negative experiences reported were to a large extent the stress the participants felt when they tried to find time to practice with the intended duration and frequency.

What I do think is that I have felt a little stress that there have been so many times when we've had, so to speak, . . . when it has been expected that you should do these exercises. It's been something that I thought, what can I say, was a bit stressful.

Woman, 26 years

Some participants found it stressful to plan for the training on their own. Somewhat related to the issue of planning was a statement regarding difficulties in choosing between different exercises in the program. Lack of contact with other participants was also brought up as a dissatisfying, in contrast to the expressed view that anonymity was a good thing.

## Discussion

### Principal Findings

This study gave support for the usability and acceptability of our self-administered Internet-based mindfulness training program among those who completed it. Although less than two-fifth of the participants completed the full program and participants practiced less than recommended, a significant increase in psychological well-being was observed. There were, however, no statistically significant differences between the intervention and control group. Most of those completing the program expressed that the program was meaningful and helpful in improving their way of being. The flexibility to perform exercises where and when it was convenient for them was appreciated and overall the program layout was perceived as satisfactory. More frequent contact with the study coordinators and reminders were suggested as ways to help participants to complete the daily exercises and to increase the likelihood of successful completion of the program. A major difficulty for several of the participants was to find enough time to practice. In addition, the majority of the participants perceived the program as challenging.

### Comparison With Prior Work

The fact that no statistically significant differences in outcomes between the mindfulness and expressive writing group was detected could be due to the active condition in the control group and the large drop-out rate in the mindfulness group. A large drop-out rate is often associated with an underestimation of the intervention effect in intention-to-treat analysis using last-observation-carried-forward approach [41-43]. The rate of participants completing the full program in this study corresponds to previous experiences from studies of Internet-based programs [22,23,29,44]. In an RCT assessing the effects of a brief 14-day Internet-based mindfulness-based intervention among British students, 43% completed a postintervention measure [23]. In another study of an Internet-based mindfulness program for stress management, the 8-week completion rate was 41% [22]. Interestingly, in this study the proportion of men who did not complete the full program was much larger than the proportion of women. Previous studies have indicated that women are more interested in participating in mindfulness training programs than men [23,45]. In a study among British students, only 12 out of 104 participants who signed up for an Internet-based mindfulness-based intervention were men [23]. However, no gender differences between the British students who completed the mindfulness-based program and those who dropped out were found. In our study, the students were not aware of which program they would be randomly assigned to when they signed up. Students might therefore have realized that they were expected to practice mindfulness after having consented to participate, increasing the risk for early termination among those who had a less positive attitude to mindfulness. The reasons for early termination of the program were not systematically explored in this study, but several of the participants stated spontaneously that lack of time was the main reason, which is in line with earlier research [22,29]. The fact that 70% of the participants in the less time-consuming and shorter expressive writing program completed their program supports that hypothesis. About a quarter of the students left the program early (before initiating week 2). Reasons for this could be that they did not like the program or realized that they did not have enough time to spend on the program. In a similar study of a brief 14-day program instructing British students to listen daily to a 10-minute guided mindfulness meditation exercise, more than half of the students dropped out before completing a postassessment questionnaire, indicating that there are other reasons than lack of time that influence termination shortly after initiation [23]. In the interviews after the program in our study, lack of contact with other participants was brought up as a negative feature of the program, similar to what has been reported in earlier research [30]. In a US study of an Internet-based mindfulness program, however, the completion rate among participants who received an Internet-based program without a message board was only slightly lower (41%) compared to participants who had access to an Internet-based message board where they could make contact with other participants (44%) [22], suggesting that contact with other participants does not contribute very much to an increased completion rate. Interviewees also mentioned the importance of contact with the study coordinators for successful completion of the program. Results from another study using computer-based mindfulness meditation training indicated that virtual coach-based training of mindfulness is both feasible and potentially more effective than a self-administered program [46]. One suggestion from the completers in this study regarding facilitation of completion of the mindfulness program was to arrange a meeting between participants and study coordinators at the beginning of the program or at least having a phone call. In an expert review on Internet-based psychological treatments for depression [47] examining different levels of contact between therapists and client in relation to outcomes, the authors stated that contact before and/or after the treatment may potentially enhance both guided and unguided Internet-based psychological treatments. Whether this is the case for self-administrated mindfulness-based and Internet-based programs among students is still unknown.

The flexibility regarding location and time for training was perceived as positive by participants, in line with earlier research [5,7,8,25]. Anonymity was also mentioned as beneficial, which has been previously observed as advantageous for participants in Internet-based interventions [6]. Furthermore, the participants reported that it was easy to navigate the program content using the Internet-based platform and that they had few or no problems with technical issues, important conditions when distributing an Internet-based program [27].

Choosing between different exercises and having to plan one’s own training were considered demanding by some of those interviewed. Perhaps some participants might have felt the need for clearer guidance and support in the latter part of the program. Earlier research has shown that participants need support, for example in managing time challenges, when taking part in Internet-based programs [25,30].

The participants did not spend the intended amount of time practicing mindfulness in this training program. It is possible that greater improvements in well-being and depression symptoms could have been reached with a higher level of compliance. However, a previous 14-day Internet-based mindfulness-based program encouraging students to practice mindfulness meditation 10 minutes each day resulted in a significant group × time interaction for anxiety/depression symptoms, suggesting that it is possible to achieve improvements in mental states with a rather limited effort. In summary, results from this study suggest that students can benefit from an Internet-based mindfulness training program.

Our study highlights the challenge of developing health-related Internet-based interventions, including mindfulness-based programs, that are sufficiently easy to complete for an intended target group. A way to enhance completion of Internet-based programs might be to use more reminders and make personal contact by phone before program start. Less time-consuming exercises and a shorter program period might also be considered in order to raise the completion rates in these interventions. Acquiring further knowledge about how to develop and deliver such programs is warranted in order to make use of the potential of the Internet in health care and other relevant settings.

### Limitations

Although this study is of great interest because it is one of the first to explore the feasibility and effect of an Internet-based mindfulness intervention, it has several limitations. First, the participants were not required to have a certain level of stress or other psychological symptoms to be included in the study, which may have reduced the ability to detect changes in the outcome measures. A possible absence of stress or distress may also have influenced participant perceptions of the program and their motivation to practice. Perhaps they would had given the program a higher priority in their daily lives if they had felt that they really needed the training. Second, our study did not systematically investigate if the students experienced some negative effects from the mindfulness training. A literature review from 2008 concluded that no negative effects from mindfulness-based interventions have been documented [48]. However, research of face-to-face treatment suggests that 5% to 10% of patients experience negative effects in terms of deterioration, and other types of negative effects have been proposed to exist as well [49]. Third, there is a risk that the method for selecting students for interviews brought bias to the results. However, some of the first students who were asked to participate in an interview did not have time for it immediately and others who finished the program later volunteered for an interview, which led to a more mixed group of interviewees. Another concern regarding bias was that one of the study coordinators interviewed the participants, potentially influencing their responses. We consider the risk of socially desirable replies to be highest concerning the issue of support from study coordinators but to be smaller regarding topics related to technique, information, instructions, and exercises, as these issues are not closely connected to the personality and skills of the coordinator. Fourth, the study was carried out among students, which limits the generalizability of the findings to other groups. Students are perhaps relatively accustomed to Web technology and may have found the program easier to navigate than for older people, for example. In addition, women were largely overrepresented in the study, especially in the investigation of usability and acceptability, which limits the generalizability of our findings to men. Another issue that limits the generalizability of the results lies in the fact that the participants received reimbursement for their participation. It is possible that the expectations regarding the program and perceived improvements of well-being would have been different without a reimbursement.

### Conclusions

Our findings support the usability and acceptability of a self-administered Internet-based mindfulness training program for several participants. The program showed potential for increasing psychological well-being. However, additional modification to the target group might be needed to increase retention and compliance.

## Abbreviations

MANOVA: multivariate analysis of variance
MBSR: mindfulness-based stress reduction
RCT: randomized controlled trial
SD: standard deviation

## Appendix

Multimedia Appendix 1 Screenshot 1.

Multimedia Appendix 2 Screenshot 2.

Multimedia Appendix 3 Screenshot 3.

Multimedia Appendix 4 CONSORT-eHealth Checklist V1.6.
