# Supplementary figures and images for: Feasibility and Outcomes of an Internet-Based Mindfulness Training Program: A Pilot Randomized Controlled Trial
Source: JMIR Ment Health. 2016 Jul 22;3(3):e33. doi: 10.2196/mental.5457 (PMC4975795; doi:10.2196/mental.5457)

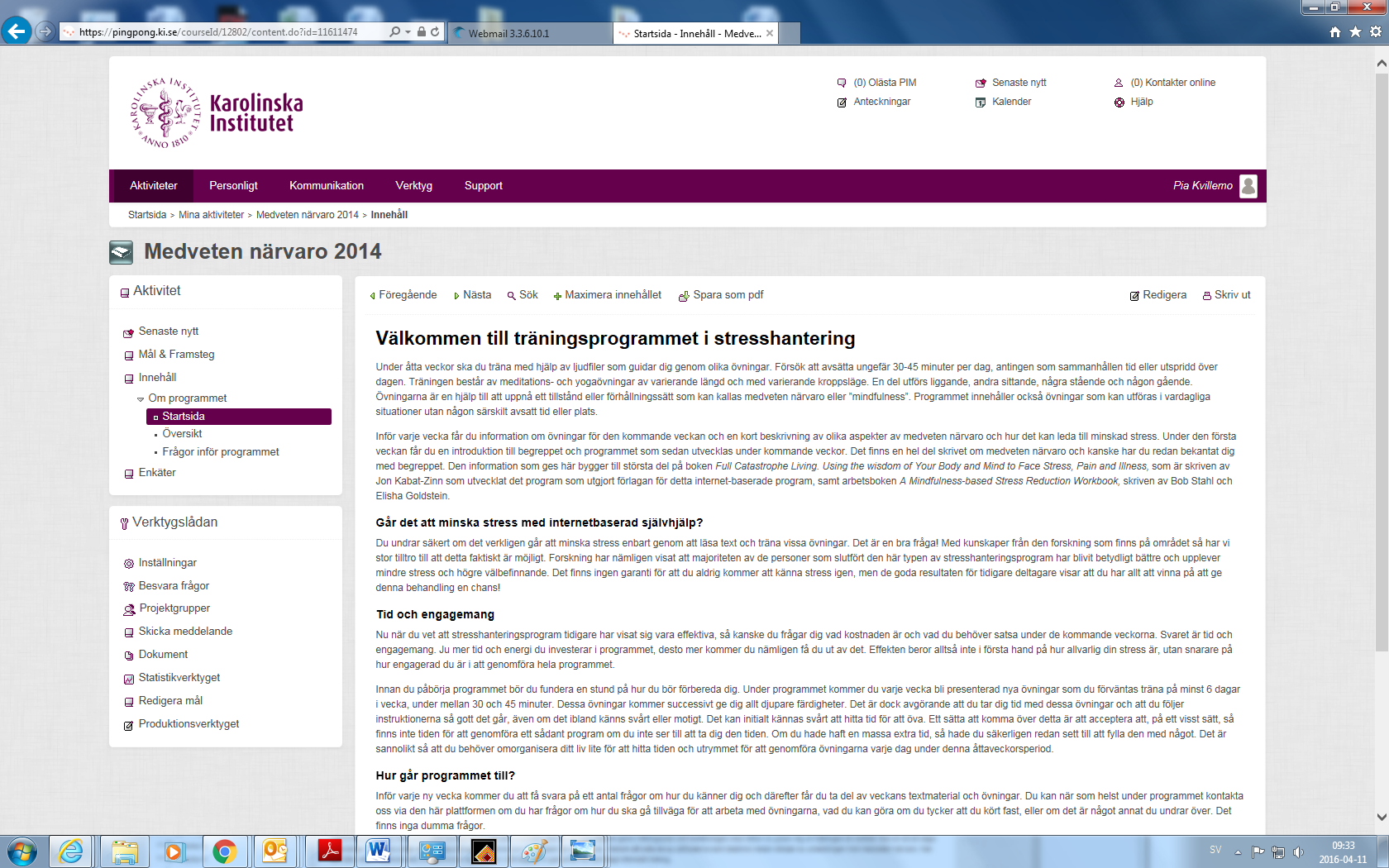

Supplement: Multimedia Appendix 1 [file mental_v3i3e33_app1.png]

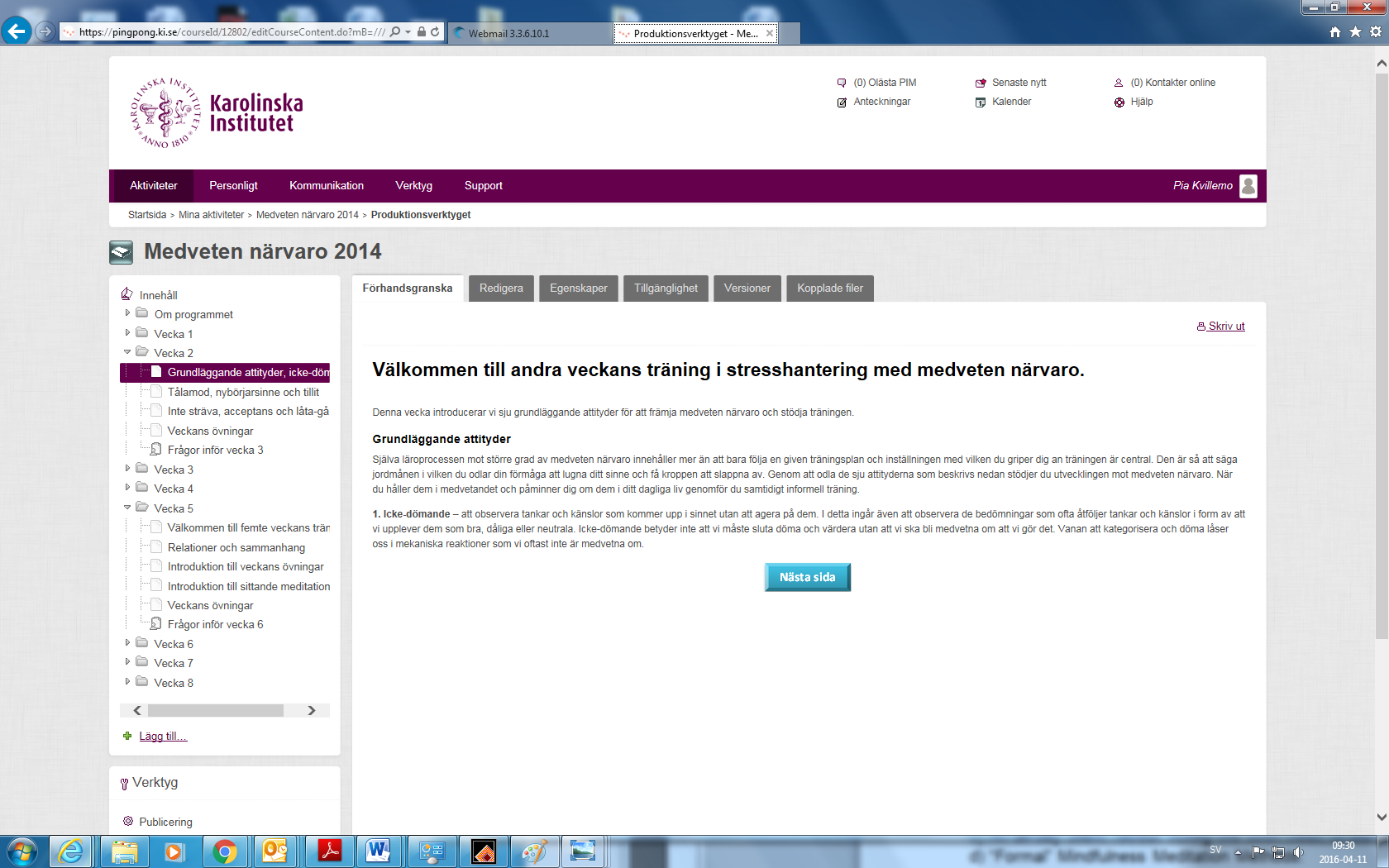

Supplement: Multimedia Appendix 2 [file mental_v3i3e33_app2.png]

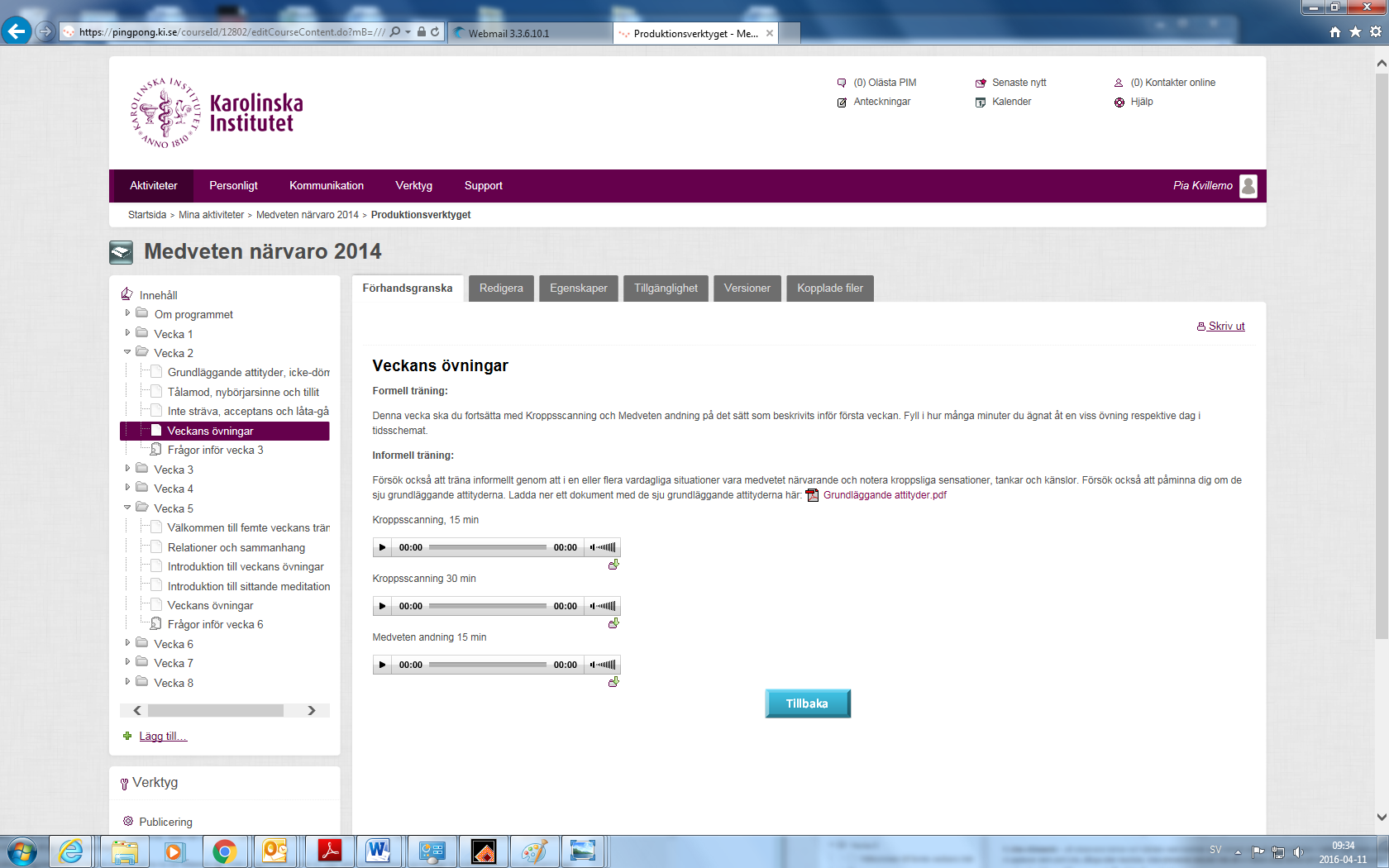

Supplement: Multimedia Appendix 3 [file mental_v3i3e33_app3.png]
